# Sharp two-parameter bounds for the identric mean

**DOI:** 10.1186/s13660-018-1917-2

**Published:** 2018-11-22

**Authors:** Omran Kouba

**Affiliations:** Department of Mathematics, Higher Institute for Applied Sciences and Technology, Damascus, Syria

**Keywords:** 26E60, 26D07, Arithmetic Mean, Geometric Mean, Harmonic Mean, Identric Mean

## Abstract

For $t\in [0,1/2]$ and $s\ge 1$, we consider the two-parameter family of means

$$ Q_{t,s}(a,b)=G^{s}\bigl(ta+(1-t)b,(1-t)a+tb\bigr)A^{1-s}(a,b), $$

where *A* and *G* denote the arithmetic and geometric means. Sharp bounds for the identric mean in terms of $Q_{t,s}$ are obtained. Our results generalize and extend bounds due to Chu et al. (Abstr. Appl. Anal. 2011:657935, 2011) and to Wang et al. (Appl. Math. Lett. 25:471–475, 2012).

## Introduction

The study of inequalities involving means has become very popular in recent years because of their applications in various kinds of areas of mathematics. Finding sharp bounds for inequalities is an important task in order to have more accurate results in the aforementioned areas.

Let us fix some notation in order to describe our results. For distinct positive real numbers *a* and *b*, we recall that the arithmetic mean $A(a,b)$, the geometric mean $G(a,b)$, the harmonic mean $H(a,b)$, and the identric mean $I(a,b)$ are respectively defined by

$$ A(a,b)=\frac{a+b}{2},\qquad G(a,b)=\sqrt{ab},\qquad H(a,b)= \frac{2ab}{a+b},\qquad I(a,b)=\frac{1}{e} \biggl( \frac{a^{a}}{b^{b}} \biggr) ^{1/(a-b)}. $$

Inequalities relating means in two variables have attracted and continue to attract the attention of mathematicians. Many articles studying the properties of means of two variables have been published, and there is a large body of mathematical literature about comparing pairs of means. The interested reader may consult [1–3, 5–7, 9–11] and the references therein.

For example, Alzer and Qui considered in [3] the following inequality relating the identric, geometric, and arithmetic means:

$$ \alpha A(a,b)+(1-\alpha )G(a,b) < I(a,b)< \beta A(a,b)+(1-\beta )G(a,b). $$

They proved that it holds, for every distinct positive numbers *a* and *b*, if and only if $\alpha \leq 2/3$ and $\beta \geq 2/e$.

This was later complemented by Trif [12] who proved that, for $p\geq 2$ and every distinct positive number *a* and *b*, we have

$$ \alpha A^{p}(a,b)+(1-\alpha )G^{p}(a,b) < I^{p}(a,b)< \beta A^{p}(a,b)+(1- \beta )G^{p}(a,b) $$

if and only if $\alpha \leq (2/e)^{p}$ and $\beta \geq 2/3$.

Similarly, it is proved in [5] that the inequality

$$ I^{p}(a,b)< \frac{2}{3}A^{p}(a,b)+\frac{1}{3}G^{p}(a,b) $$

holds true for every distinct positive number *a* and *b* if and only if $p\geq \ln ( \frac{3}{2} ) /\ln ( \frac{e}{2} ) \approx 1.3214$, and that the reverse inequality holds true for every distinct positive number *a* and *b* if and only if $p\leq 6/5= 1.2$, this generalizes the results of [8] and [12].

In this paper we continue the search for nontrivial bounds for the identric mean by studying a new family of two parameter means of two variables. The article is organized as follows. In Sect. 2 we present and discuss the main results. Section 3 is devoted to the proof of several technical lemmas that will be useful for the proof of the main theorems, and in Sect. 4 the main results are discussed and proved.

## Results and discussion

Motivated by the works [4] and [13], we consider the two parameter family of means $Q_{t,s}(a,b)$ defined for $s\geq 1$, $t\in [0,1/2]$, and any positive real numbers *a* and *b* by

1 $$ Q_{t,s}(a,b)=G^{s}\bigl(t a+(1-t)b,tb+(1-t)a \bigr)A^{1-s}(a,b). $$

Indeed, the authors in [4, 13] compare the identric mean to

$$\begin{aligned} Q_{t,2}(a,b) =H\bigl(t a+(1-t)b,tb+(1-t)a\bigr), \end{aligned}$$

see [4, Theorem 1.1], and to

$$\begin{aligned} Q_{t,1}(a,b) =G\bigl(t a+(1-t)b,tb+(1-t)a\bigr), \end{aligned}$$

see [13, Theorem 1.1].

The aim of this work is to produce a general result comparing the identric mean to members of the family $(Q_{t,s})_{t\in [0,1/2]\times [1,+\infty )}$, which generalizes the results of [4] and [13].

Indeed, in Corollary 3.1 we prove that, for distinct positive real numbers *a* and *b* and given $s\ge 1$, the function $t\mapsto Q _{t,s}(a,b)$ is continuous and increasing. Further, for $s\geq 1$ and every distinct positive number *a* and *b*, we have

$$ Q_{0,s}(a,b)\leq Q_{0,1}(a,b)=G(a,b)< I(a,b)< A(a,b)=Q_{1/2,s}(a,b). $$

Thus, for given $s\geq 1$, it is natural to consider the sets

$$\begin{aligned} \mathcal{L}_{s} &= \bigl\{ t\in [0,1/2]: \mbox{ for all positive } a, b\mbox{ with }a\ne b, Q_{t,s}(a,b)< I(a,b) \bigr\} , \\ \mathcal{U}_{s} &= \bigl\{ t\in [0,1/2]:\mbox{ for all positive } a, b\mbox{ with }a\ne b, I(a,b)< Q_{t,s}(a,b) \bigr\} . \end{aligned}$$

Because $t\mapsto Q_{t,s}(a,b)$ is increasing, it is clear that $\mathcal{L}_{s}$ and $\mathcal{U}_{s}$ are both intervals. In Theorem 4.1 we prove that, for $s\geq 1$, $\mathcal{L}_{s}=[0,p _{s}]$, and $\mathcal{U}_{s}=[q_{s},1/2]$, where

$$ p_{s}=\frac{1}{2}-\frac{1}{2} \sqrt{1- \biggl( \frac{2}{e} \biggr) ^{2/s}} \quad \textrm{and}\quad q _{s}= \frac{1}{2}-\frac{1}{2\sqrt{3s}}. $$

This extends the results of Chu et al. [4] and Wang et al. [13].

## Preliminaries

The following lemmas pave the way to the main theorem. In Lemma 3.1 we study a family of functions using simple methods from classical analysis.

### Lemma 3.1

*For* $s\geq 1$ *and* $u\in [0,1]$, *we consider the real function* $f_{u,s}$ *defined on* $[0,1)$ *by*

2 $$ f_{u,s}(x)=1-\frac{1}{2x}\ln \biggl( \frac{1+x}{1-x} \biggr) -\frac{1}{2} \ln \bigl(1-x^{2}\bigr)+ \frac{s}{2}\ln \bigl(1-ux^{2}\bigr). $$

- *The necessary and sufficient condition to have* $f_{u,s}(x)>0$ *for* $x\in (0,1)$ *is that* $3su\leq 1$.
- *The necessary and sufficient condition to have* $f_{u,s}(x)<0$ *for* $x\in (0,1)$ *is that* $u+(2/e)^{2/s}\geq 1$.

### Proof

We consider only the case $u\in (0,1]$, since $f_{0,s}$ is independent of *s* and positive on $(0,1)$. It is straightforward to see that $f_{u,s}^{\prime }(x)=h_{u,s}(x)/x^{2}$, where

$$ h_{u,s}(x)=-x+\frac{1}{2}\ln \biggl( \frac{1+x}{1-x} \biggr) - \frac{s u x ^{3}}{1-u x^{2}}, $$

and that

$$ h_{u,s}^{\prime }(x)=\frac{x^{2}}{(1-x^{2})(1-ux^{2})^{2}}T_{u,s}\bigl(x ^{2}\bigr), $$

where $T_{u,s}$ is the trinomial defined by

$$ T_{u,s}(X)=(1-s)u^{2}X^{2}-(2-3 s-s u)uX+(1-3 s u). $$

Noting that $T_{u,s}(1)=(1-u)^{2}\geq 0$ and $T_{u,s}(0)=1-3su$, we see that we have two cases:

- First, $T_{u,s}(0)\geq 0$, or equivalently $3su\leq 1$. Again, we distinguish two cases:
  ∘: If $s=1$, then clearly the zero of $T_{u,1}$ does not belong to $(0,1)$ and $T_{u,s}$ has a positive sign on $(0,1)$.
  ∘: If $s>1$, then the coefficient of $X^{2}$ in $T_{u,s}$ is negative, and the fact that both $T_{u,s}(0)$ and $T_{u,s}(1)$ are nonnegative implies that the zeros $z_{0}< z_{1}$ of $T_{u,s}$ satisfy the inequality $z_{0}\leq 0<1\leq z_{1}$. Hence, $T_{u,s}$ has a positive sign on $(0,1)$ in this case also.
  It follows that in this case $h_{u,s}$ is increasing on $[0,1)$. But $h_{u,s}(0)=0$, so $h_{u,s}$ is positive on $(0,1)$. Therefore $f_{u,s}$ is increasing on $(0,1)$. Finally, the fact that $\lim_{x\to 0^{+}}f_{u,s}(x)=0$ implies that $f_{u,s}(x)>0$ for every $x\in (0,1)$ in this case.
- Second, $T_{u,s}(0)<0$, or equivalently $3su> 1$. This means that $T_{u,s}$ has a *unique* zero $z_{0}$ in the interval $(0,1]$ (because $\deg (T_{u,s})\leq 2$).
  ∘: If $u=1$, then $z_{0}=1$ and $h_{1,s}$ is decreasing on $[0,1]$. But $h_{1,s}(0)=0$, so $h_{1,s}$ is negative on $(0,1)$. This shows that $f_{1,s}$ is decreasing on $(0,1)$. Finally, we have $\lim_{x\to 0^{+}}f_{1,s}(x)=0$, and consequently $f_{1,s}(x)<0$ for every $x\in (0,1)$.
  ∘: If $u<1$, then $z_{0}\in (0,1)$. So $h_{u,s}$ is decreasing on $[0,z_{0}]$ and increasing on $[z_{0},1]$. But $h_{u,s}(0)=0$ so $h_{u,s}(z_{0})<0$. On the other hand $\lim_{x\to 1^{-}}h_{u,s}(x)=+\infty $. So there exists a unique real number $y_{0}\in (z_{0},1)$ such that $h_{u,s}(y_{0})=0$. Thus $h_{u,s}(x)<0$ for $x\in (0,y_{0})$ and $h_{u,s}(x)>0$ for $x\in (y_{0},1)$. This implies that $f_{u,s}$ is decreasing on $(0,y_{0})$ and increasing on $(y_{0},1)$. Finally we have $\lim_{x\to 0^{+}}f_{u,s}(x)=0$ and $\lim_{x\to 1^{-}}f_{u,s}(x)= \ln ( e(1-u)^{s/2}/2 ) $.
  This shows that the necessary and sufficient condition for $f_{u,s}$ to be negative on $(0,1)$ is that either $u=1$ or $u<1$ and $\ln ( e(1-u)^{s/2}/2 ) \leq 0$ which is equivalent to the condition $1\leq u+(2/e)^{2/s}$.

This achieves the proof of Lemma 3.1. □

Next we introduce the set ${\mathcal{D}}$ defined as follows:

$$ {\mathcal{D}}= \bigl\{ (a,b)\in \mathbb {R}^{2}:a>b>0 \bigr\} . $$

It is sufficient to consider couples $(a,b)$ from ${\mathcal{D}}$, since the considered means are symmetric functions of their arguments. Lemma 3.2 explains why the family of functions studied in Lemma 3.1 is important to our study.

### Lemma 3.2

*Consider* $(a,b)\in {\mathcal{D}}$ *and let* $v=\frac{a-b}{a+b}$.

- *For* $s\geq 1$ *and* $t\in [0,1/2]$, *we have*
  $$ \ln \biggl( \frac{Q_{t,s}(a,b)}{A(a,b)} \biggr) =\frac{s}{2}\ln \bigl( 1-(1-2t)^{2} v^{2} \bigr) . $$
- *Also*, *for the identric mean*, *we have*
  $$ \ln \biggl( \frac{I(a,b)}{A(a,b)} \biggr) =-1+\frac{1}{2}\ln \bigl(1-v^{2}\bigr)+ \frac{1}{2v}\ln \biggl( \frac{1+v}{1-v} \biggr) . $$

### Proof

Indeed, (a) follows from the simple fact that

$$ G\bigl(ta+(1-t)b,tb+(1-t)a\bigr)=A(a,b)\sqrt{1-(1-2t)^{2} \biggl( \frac{a-b}{a+b} \biggr) ^{2}}. $$

To see (b), we note that

$$\begin{aligned} \frac{I(a,b)}{A(a,b)} &=\frac{1}{e}\frac{2}{a+b}a^{a/(a-b)}b^{-b/(a-b)} =\frac{1}{e} \biggl( \frac{2a}{a+b} \biggr) ^{\frac{a}{a-b}} \biggl( \frac{2b}{a+b} \biggr) ^{\frac{-b}{a-b}} \\ &=\frac{1}{e} \biggl( 1+\frac{a-b}{a+b} \biggr) ^{\frac{1}{2}+ \frac{a+b}{2(a-b)}} \biggl( 1-\frac{a-b}{a+b} \biggr) ^{\frac{1}{2}- \frac{a+b}{2(a-b)}}=\frac{1}{e} (1+v )^{\frac{1+v}{2v}} (1-v )^{ \frac{v-1}{2v}}. \end{aligned}$$

This concludes the proof of Lemma 3.2. □

The next corollary is an immediate consequence of part (a) of Lemma 3.2.

### Corollary 3.1

*For distinct positive real numbers* *a* *and* *b*, *and for given* $s\ge 1$, *the function* $t\mapsto Q_{t,s}(a,b) $ *is continuous and increasing on the interval* $[0,1/2]$.

### Remark 3.1

Combining (a) and (b) from Lemma 3.2, we see immediately that if $f_{u,s}$ is the function defined in Lemma 3.1 then, for every $(a,b)\in {\mathcal{D}}$, we have

$$ \ln \biggl( \frac{Q_{t,s}(a,b)}{I(a,b)} \biggr) =f_{(1-2t)^{2},s} \biggl( \frac{a-b}{a+b} \biggr) . $$

This explains the importance of the family of functions in Lemma 3.1 to our study.

## The main theorem

In this section we prove our main result which states that, for $s\ge 1$ and $u,v\in [0,1/2]$, the double inequality $Q_{u,s}(a,b)< I_{(}a,b)< Q_{v,s}(a,b)$ holds for all distinct positive real numbers *a* and *b* if and only if

$$ u\le \frac{1}{2}-\frac{1}{2}\sqrt{1- \biggl( \frac{2}{e} \biggr) ^{2/s}} \quad \textrm{and}\quad v\ge \frac{1}{2}- \frac{1}{2\sqrt{3s}}. $$

Further, this result is used to obtain in Corollary 4.1 an upper bound counterpart of the inequality due to Seiffert [11] about the ratio $A(a,b)/I(a,b)$.

### Theorem 4.1

*Let* *s* *be a real number such that* $s\geq 1$, *and define the sets*

$$\begin{aligned}& {\mathcal{L}}_{s} = \bigl\{ t\in [0,1/2]: \forall (a,b)\in { \mathcal{D}},~ Q_{t,s}(a,b)< I(a,b) \bigr\} , \\& {\mathcal{U}}_{s} = \bigl\{ t\in [0,1/2]: \forall (a,b)\in { \mathcal{D}},~ I(a,b)< Q_{t,s}(a,b) \bigr\} . \end{aligned}$$

*Then*

$$ {\mathcal{L}}_{s}= \biggl[ 0,\frac{1}{2}-\frac{1}{2} \sqrt{1- \biggl( \frac{2}{e} \biggr) ^{2/s}} \biggr] \quad\textit{and}\quad {\mathcal{U}}_{s}= \biggl[ \frac{1}{2}- \frac{1}{2 \sqrt{3s}},\frac{1}{2} \biggr] . $$

### Proof

First note that

$$ \biggl\{ \frac{a-b}{a+b}:(a,b)\in {\mathcal{D}} \biggr\} = (0,1 ). $$

So, using Corollary 3.1, we see that $t\in {\mathcal{L}}_{s}$ if and only if $f_{(1-2t)^{2},s}(x)<0$ for every $x\in (0,1)$. However, according to Lemma 3.1, this is equivalent to $(1-2t)^{2}+(2/e)^{2/s} \geq 1$ or $(1-\sqrt{1-(2/e)^{2/s}})/2\geq t$. This proves that

$$ \mathcal{ L}_{s}= \biggl[ 0,\frac{1}{2}-\frac{1}{2} \sqrt{1- \biggl( \frac{2}{e} \biggr) ^{2/s}} \biggr] . $$

Similarly, using Corollary 3.1, we see that $t\in {\mathcal{U}} _{s}$ if and only if $f_{(1-2t)^{2},s}(x)>0$ for every $x\in (0,1)$. Again, by Lemma 3.1, this is equivalent to $3s(1-2t)^{2}\leq 1$ or $(1-1/\sqrt{3s})/2\leq t$. This proves

$$ \mathcal{U}_{s}= \biggl[ \frac{1}{2}-\frac{1}{2\sqrt{3s}}, \frac{1}{2} \biggr] , $$

and achieves the proof of Theorem 4.1. □

When $s=2$, the definition of $Q_{t,s}(a,b)$ given by (1) is reduced to the harmonic mean of $ta+(1-t)b$ and $(1-t)a+tb$. So Theorem 4.1 yields in this case the following theorem from [4].

### Theorem 4.2

([4])

*The necessary and sufficient condition on* *p*, *q* *from* $[0,1/2]$ *to have*

$$ H\bigl(pa+(1-p)b,pb+(1-p)a\bigr)< I(a,b)< H\bigl(qa+(1-q)b,qb+(1-q)a\bigr) $$

*for every distinct positive number* *a* *and* *b* *is that*

$$ p\leq \frac{1-\sqrt{1-2/e}}{2}\quad \textit{and}\quad q\geq \frac{6- \sqrt{6}}{12}. $$

Similarly, when $s=1$, the definition of $Q_{t,s}(a,b)$ given by (1) is reduced to the geometric mean of $ta+(1-t)b$ and $(1-t)a+tb$. So Theorem 4.1 yields in this case the following theorem from [13].

### Theorem 4.3

([13])

*The necessary and sufficient condition on* *p*, *q* *from* $[0,1/2]$ *to have*

$$ G\bigl(pa+(1-p)b,pb+(1-p)a\bigr)< I(a,b)< G\bigl(qa+(1-q)b,qb+(1-q)a\bigr) $$

*for every distinct positive number* *a* *and* *b* *is that*

$$ p\leq \frac{1-\sqrt{1-4/e^{2}}}{2}\quad \textit{and}\quad q \geq \frac{3-\sqrt{3}}{6}. $$

In the next corollary, the lower bound is an inequality due to Seiffert [11], it appears also in [10], while the upper bound is new and to be compared with the results of Sándor and Trif in [10].

### Corollary 4.1

*For every positive number* *a* *and* *b*, *we have*

$$ \exp \biggl( \frac{1}{6} \biggl( \frac{a-b}{a+b} \biggr) ^{2} \biggr) \leq \frac{A(a,b)}{I(a,b)}\leq \exp \biggl( \biggl( \ln \frac{e}{2} \biggr) \biggl( \frac{a-b}{a+b} \biggr) ^{2} \biggr) . $$

### Proof

Indeed, for $s\geq 1$, let

$$ p_{s}=\frac{1}{2}-\frac{1}{2} \sqrt{1- \biggl( \frac{2}{e} \biggr) ^{2/s}} \quad \textit{and}\quad q _{s}=\frac{1}{2}-\frac{1}{2\sqrt{3s}}. $$

Using Theorem 4.1, for every $(a,b)\in {\mathcal{D}}$, we have

$$ Q_{p_{s},s}(a,b)< I(a,b)< Q_{q_{s},s}(a,b). $$

This can be written as

$$ \frac{A(a,b)}{Q_{q_{s},s}(a,b)}< \frac{A(a,b)}{I(a,b)}< \frac{A(a,b)}{Q _{p_{s},s}(a,b)}. $$

Using Lemma 3.2, this is equivalent to

$$ \biggl( 1-\frac{v^{2}}{3s} \biggr) ^{-s/2}< \frac{A(a,b)}{I(a,b)} < \biggl( 1- \biggl( 1- \biggl(\frac{2}{e} \biggr)^{2/s} \biggr) v^{2} \biggr) ^{-s/2}, $$

where $v=(a-b)/(a+b)$. Now, letting *s* tend to +∞, we obtain

$$ e^{v^{2}/6}\leq \frac{A(a,b)}{I(a,b)}\leq e^{ ( \ln \frac{e}{2} ) v ^{2}}, $$

which is the conclusion of Corollary 4.1. □

In fact, because of the “limit argument” in the proof of Corollary 4.1, we lost the strict inequalities for distinct positive real arguments. However, studying the family of functions $(g_{t})_{t\in (0,+\infty )}$ defined by

$$ g_{t}(x)=1-\frac{1}{2x}\ln \biggl( \frac{1+x}{1-x} \biggr) - \frac{1}{2} \ln \bigl(1-x^{2}\bigr)-t x^{2}, $$

using similar arguments to those used in Lemma 3.1, we can prove the following exact version of Corollary 4.1, which extends the results of Seiffert [11] and those of Sándor and Trif [10].

### Theorem 4.4

*The necessary and sufficient condition on* *p*, *q* *from* $(0,+\infty )$ *to have*

$$ \forall (a,b)\in {\mathcal{D}},\quad \exp \biggl( p \biggl( \frac{a-b}{a+b} \biggr) ^{2} \biggr) < \frac{A(a,b)}{I(a,b)} < \exp \biggl( q \biggl( \frac{a-b}{a+b} \biggr) ^{2} \biggr) $$

*is that* $p\leq \frac{1}{6}$ *and* $q\geq \ln (\frac{e}{2})$.

## Conclusion

In this work, we have considered a new two-parameter family of means, and we have compared them to the identric mean giving sharp upper and lower bounds.
